# Supplementary material for: Estimation of substitution and indel rates via k-mer statistics
Source: Algorithms Bioinform. Author manuscript; Available in PMC 2026 Apr 22. (PMC13098707; doi:10.4230/LIPIcs.WABI.2025.16)
Supplement: 1 [file NIHMS2163779-supplement-1.pdf]

A List of notations used in the manuscript

For convenience, we gather in the following table the notation used in the manuscript.

Notation	Meaning
p_s	Substitution rate
p_d	Deletion rate
d	Average insertion length
S	The original string
S'	The mutated string
L	Length of (number of characters in) S
L'	Length of (number of characters in) S'
f_A, f_C, f_G, f_T	Number of 'A', 'C', 'G', 'T' in S, respectively. Here, $f_A + f_C + f_G + f_T = L$.
f_A', f_C', f_G', f_T'	Number of 'A', 'C', 'G', 'T' in S', respectively. Here, $f_A' + f_C' + f_G' + f_T' = L'$.
P	$P = L' - 4f_A'$
$\mathcal{K}$	Number of k-spans in S. Here, $\mathcal{K} = L - k + 1$.
$\mathcal{K}'$	Number of k-spans in S'. Here, $\mathcal{K}' = L' - k + 1$.
S_i	The character in S at position i
Q_i	The string inserted after S_i by the mutation process
l_i	Length of Q_i
$\mathcal{K}_i$	The interval $[i, i + k - 1]$, inclusive of the endpoints
$\mathcal{N}$	Number of k-spans that were not mutated by the mutation process
$\mathcal{S}$	Number of k-spans where a single substitution, and no other mutation was introduced by the mutation process
$\mathcal{D}$	Number of k-spans where a single deletion, and no other mutation was introduced by the mutation process
$\mathcal{I}$	Number of k-spans where a single insertion, and no other mutation was introduced by the mutation process
$\mathcal{L}_{\text{ins}}$	Total number of characters inserted by the mutation process.
$\mathcal{L}_{\text{del}}$	Total number of characters deleted by the mutation process.

Table 1 List of notations.

B Preliminaries

This section gathers several definitions and standard concentration inequalities that we use in our proofs. For a random variable X with finite non-zero variance σ^2, Chebyshev's inequality says that for any real value $r > 0$

$$\Pr\left[|X - \mathbf{E}[X]| \geq r\sigma\right] \leq \frac{1}{r^2}.$$

Sums of independent random variables can exhibit sharper concentration, and this is captured by the following standard inequalities. The first one is the Chernoff bound for sums of independent and identically distributed Bernoulli random variables [1, 4].

▶ **Lemma 10.** *Let $X_1, \ldots, X_n$ be a sequence of independently and identically distributed Bernoulli random variables. Consider $X = \sum_{i=1}^{n} X_i$ and let $\mathbf{E}[X] = \mu$. Then, the following three inequalities*

hold:

$$\Pr(X \geq (1+\delta)\mu) \leq e^{-\delta^2\mu/(2+\delta)}, \qquad 0 \leq \delta.$$

$$\Pr(X \leq (1-\delta)\mu) \leq e^{-\delta^2\mu/2}, \qquad 0 < \delta < 1. \tag{13}$$

$$\Pr(|X-\mu| \geq \delta\mu) \leq 2e^{-\delta^2\mu/3}, \qquad 0 < \delta < 1. \tag{14}$$

A more general inequality that applies to sums of independent and bounded random variables is Hoeffding's inequality [11].

▶ **Lemma 11.** *Let $X_1, \ldots, X_n$ be a sequence of independently and identically distributed random variables taking values in $[a,b]$. Let $X = \sum_{i=1}^n X_i$, and $\mathbf{E}[X] = \mu$. Then, for any $\delta > 0$:*

$$\Pr(|X-\mu| \geq \delta) \leq 2e^{-\frac{2\delta^2}{n(b-a)^2}}.$$

We will also analyze sums of certain *unbounded* random variables. For this, we recall the definition of sub-exponential random variables. For a comprehensive survey about this type of random variables, the reader is referred to [23, Chapter 2.7].

▶ **Definition 12.** *A random variable X such that $\mathbf{E}[X] = 0$ is called sub-exponential if either of the following equivalent properties holds:*

1. *There exists $J_1 > 0$ such that for all $t \geq 0$:*

$$\Pr\Big[|X| \geq t\Big] \leq 2\exp\{-t/J_1\}.$$

2. *There exists $J_2 > 0$ such that*

$$\mathbf{E}\Big[e^{|X|/J_2}\Big] \leq 2. \tag{15}$$

The constants J_1 and J_2 differ by an absolute multiplicative constant. The smallest J_2 such that (15) holds is called the *sub-exponential norm of X* and is denoted by $\|X\|_{\psi_1}$. For sums of sub-exponential random variables, the following version of Bernstein's inequality is known; see [23, Theorem 2.8.1].

▶ **Lemma 13.** *Let $X_1, \ldots, X_n$ be a sequence of independent, mean-zero, sub-exponential random variables, and let $X = \sum_{i=1}^n X_i$. Then, there exists an absolute constant $c > 0$ such that for every $t \geq 0$:*

$$\Pr[|X| \geq t] \leq 2\exp\left[-c\min\left(\frac{t^2}{\sum_{i=1}^N \|X_i\|_{\psi_1}^2}, \frac{t}{\max_i \|X_i\|_{\psi_1}}\right)\right].$$

C Proofs of preliminary statistics

We prove in this section the lemmas from Section 3.

▶ **Lemma 1.** *Let L' be the length of S'. Then*

$$\mathbf{E}[L'] = L\,(1 + d - p_d), \tag{1}$$

$$\mathbf{Var}[L'] = L\Big(d(d+1) + p_d(1-p_d)\Big).$$

Proof. For $1 \leq i \leq L$, let D_i be an indicator random variable defined as:

$$D_i = \begin{cases} 1 & \text{if character } S_i \text{ is removed,} \\ 0 & \text{otherwise.} \end{cases}$$

Using the D_i's and l_i's, we can express L' as:

$$L' = L + \sum_{i=1}^{L} l_i - \sum_{i=1}^{L} D_i, \tag{16}$$

and the linearity of expectation yields that

$$\mathbf{E}[L'] = L + \sum_{i=1}^{L} \mathbf{E}[l_i] - \sum_{i=1}^{L} \mathbf{E}[D_i].$$

We note that l_i is a geometric distribution with mean of d; i.e., $\mathbf{E}[l_i] = d$. Moreover, $\mathbf{E}[D_i] = \Pr[D_i = 1] = p_d$, and thus we obtain that $\mathbf{E}[L'] = L(1 + d - p_d)$ as claimed.

To compute the variance of L', note that D_i's and l_i's are all independent. Hence,

$$\mathbf{Var}[L'] = \mathbf{Var}\Big[L + \sum_{i=1}^{L} l_i - \sum_{i=1}^{L} D_i\Big] = \sum_{i=1}^{L} \mathbf{Var}[l_i] - \sum_{i=1}^{L} \mathbf{Var}[D_i].$$

The D_i's are Bernoulli random variables, and therefore, $\mathbf{Var}[D_i] = p_d(1 - p_d)$. Moreover, the l_i's are geometrically distributed with mean d, so $\mathbf{Var}[l_i] = d(d + 1)$. This yields that $\mathbf{Var}[L'] = L(d(d + 1) + p_d(1 - p_d))$. ◀

▶ **Lemma 2.** *Let f_A and $f_A{}'$ be the number of 'A's in S and S', respectively. Then,*

$$\mathbf{E}[f_A{}'] = f_A(1 - p_s - p_d) + \frac{p_s(L - f_A)}{3} + \frac{dL}{4}. \tag{2}$$

Proof. Let A_i be an indicator defined as:

$$A_i = \begin{cases} 1 & \text{if } a_i \text{ was a stay operation,} \\ 0 & \text{otherwise.} \end{cases}$$

Note that $\mathbf{E}[A_i] = \Pr[A_i = 1] = 1 - p_s - p_d$. Let us also define B_i as :

$$B_i = \begin{cases} 1 & \text{if } a_i \text{ was a substitute operation and } S'_i = \text{'A'}, \\ 0 & \text{otherwise,} \end{cases}$$

and observe that $\mathbf{E}[B_i] = \Pr[B_i = 1] = p_s/3$.

Finally, let C_i be the number of 'A's in Q_i. Then,

$$\mathbf{E}[C_i] = \sum_{\ell \geq 0} \mathbf{E}[C_i \mid l_i = \ell] \Pr[l_i = \ell] = \sum_{\ell \geq 0} \frac{\ell}{4} \Pr[l_i = \ell] = \frac{d}{4}. \tag{17}$$

We can express $f_A{}'$ as:

$$f_A{}' = \sum_{i:S_i = \text{'A'}} A_i + \sum_{i:S_i \neq \text{'A'}} B_i + \sum_{i=1}^{L} C_i, \tag{18}$$

and thus

$$\mathbf{E}[f_A{}'] = \sum_{i:S_i = \text{'A'}} \mathbf{E}[A_i] + \sum_{i:S_i \neq \text{'A'}} \mathbf{E}[B_i] + \sum_{i=1}^{L} \mathbf{E}[C_i] = f_A(1 - p_s - p_d) + \frac{(L - f_A)p_s}{3} + \frac{dL}{4},$$

as claimed. ◀

▶ **Lemma 3.** $\mathbf{E}[\mathcal{N}] = \mathcal{K}\,(1-p_s-p_d)^{\,k}\,\frac{1}{(d+1)^{\,k-1}}$.

Proof. Let N_i be the indicator variable that equals 1 if the k-span $\mathcal{K}_i$ has no mutations. Then, $\mathcal{N} = \sum_{i=1}^{\mathcal{K}} N_i$. For a k-span to have no mutations (i.e., $N_i = 1$), the mutation model would have to choose the stay operation for all of its k positions, the probability of which is $(1-p_s-p_d)^k$. Additionally, for all the $k-1$ insert positions, the mutation model would need to choose strings of length zero. Therefore, $\Pr[N_i = 1] = (1-p_s-p_d)^k/(d+1)^{k-1}$, and thus

$$\mathbf{E}[\mathcal{N}] = \sum_{i=1}^{\mathcal{K}} \Pr[N_i = 1] = \mathcal{K}\frac{(1-p_s-p_d)^k}{(d+1)^{k-1}},$$

as claimed. ◀

▶ **Lemma 4.** *The expectations of $\mathcal{S}$, $\mathcal{D}$, and $\mathcal{I}$ are given by:*

$$\mathbf{E}[\mathcal{S}] = \mathcal{K}\,k\,(1-p_s-p_d)^{k-1}\,p_s\,\frac{1}{(d+1)^{\,k-1}}. \tag{3}$$

$$\mathbf{E}[\mathcal{D}] = \mathcal{K}\,k\,(1-p_s-p_d)^{k-1}\,p_d\,\frac{1}{(d+1)^{\,k-1}}. \tag{4}$$

$$\mathbf{E}[\mathcal{I}] = \mathcal{K}\,(k-1)\,(1-p_s-p_d)^{k}\,\frac{d}{(d+1)^{\,k}}. \tag{5}$$

Proof. Let E_i be an indicator random variable defined as follows for $1 \le i \le \mathcal{K}$:

$$E_i = \begin{cases} 1 & \text{if the } k\text{-span } \mathcal{K}_i \text{ has a single substitution and no other mutation,} \\ 0 & \text{otherwise.} \end{cases}$$

We note that for a k-span to have a single substitution and no other mutations, the mutation process needs to insert 0 length strings in all the $k-1$ insert positions, and other than a single substitution position, for all other $k-1$ positions in the k-span, the mutation process needs to select a stay operation. The single substitution operation could be at any of the k positions. Then,

$$\mathbf{E}[E_i] = \frac{k(1-p_s-p_d)^{k-1}p_s}{(d+1)^{k-1}}.$$

Since $\mathcal{S} = \sum_{i=1}^{\mathcal{K}} E_i$, we obtain that

$$\mathbf{E}[\mathcal{S}] = \sum_{i=1}^{\mathcal{K}} \mathbf{E}[E_i] = \mathcal{K}k(1-p_s-p_d)^{k-1}p_s/(d+1)^{k-1}.$$

The expectations of $\mathcal{D}$ and $\mathcal{I}$ can be derived following an analogous argument; the details are omitted. ◀

D Proofs of concentration results

We prove in this section the results from Section 5.

▶ **Lemma 5.** *For any $\delta \in (0,1)$:*

$$\Pr\left[\left|\mathcal{N} - \mathbf{E}[\mathcal{N}]\right| \ge \delta\mathbf{E}[\mathcal{N}]\right] \le 3k\exp\left\{-\frac{\delta^2\mathbf{E}[\mathcal{N}]}{3k}\right\}.$$

Proof. As in the proof of Lemma 3, let N_i be the indicator variable that equals 1 if the k-span $\mathcal{K}_i$ has no mutations so that $\mathcal{N} = \sum_{i=1}^{\mathcal{K}} N_i$. Recall that $\Pr[N_i = 1] = (1 - p_s - p_d)^k/(d+1)^{k-1} = q$. The N_i's are not independent, but N_i and N_j are independent if $|i - j| \geq k$. Then, for $\ell \in \{1, \ldots, k\}$ let

$$M_\ell = \sum_{j\,:\,\ell+kj \leq L-k+1} N_{\ell+kj}.$$

Observe that the M_ℓ's are defined such that each is a sum of independent Bernoulli random variables with success probability q. The Chernoff bound in (14) implies that for $\delta \in (0, 1)$:

$$\Pr\Big[\big|M_\ell - \mathbf{E}[M_\ell]\big| \geq \delta\mathbf{E}[M_\ell]\Big] \;\leq\; 2\exp\Big\{-\frac{\delta^2\mathbf{E}[M_\ell]}{3}\Big\},$$

and this holds for $\ell \in \{1, 2, 3, \ldots, k\}$. By the triangle inequality:

$$\big|\mathcal{N} - \mathbf{E}[\mathcal{N}]\big| = \Big|\sum_{\ell=1}^{k} M_\ell - \mathbf{E}[M_\ell]\Big| \leq \sum_{\ell=1}^{k}\big|M_\ell - \mathbf{E}[M_\ell]\big|,$$

so if $\big|M_\ell - \mathbf{E}[M_\ell]\big| < \delta\mathbf{E}[M_\ell]$ for each ℓ, we obtain that $\big|\mathcal{N} - \mathbf{E}[\mathcal{N}]\big| < \delta\mathbf{E}[\mathcal{N}]$. A union bound then implies that

$$\Pr\Big[\big|\mathcal{N} - \mathbf{E}[\mathcal{N}]\big| \geq \delta\mathbf{E}[\mathcal{N}]\Big] \;\leq\; 2k\exp\Big\{-\frac{\delta^2 \min_\ell \mathbf{E}[M_\ell]}{3}\Big\}.$$

Finally, observe that $\mathbf{E}[M_\ell] \geq \lfloor (L-k+1)/k \rfloor q \geq \mathbf{E}[\mathcal{N}] - q$. Since $e^{q\delta^2/3} \leq 1.5$, we obtain that

$$\Pr\Big[\big|\mathcal{N} - \mathbf{E}[\mathcal{N}]\big| \geq \delta\mathbf{E}[\mathcal{N}]\Big] \;\leq\; 3k\exp\Big\{-\frac{\delta^2\mathbf{E}[\mathcal{N}]}{3}\Big\},$$

as claimed. ◀

▶ **Lemma 6.** *For any $\delta \in (0, 1)$:*

$$\Pr\Big[\big|\mathcal{D} - \mathbf{E}[\mathcal{D}]\big| \geq \delta\mathbf{E}[\mathcal{D}]\Big] \;\leq\; 3k\exp\Big\{-\frac{\delta^2\mathbf{E}[\mathcal{D}]}{3k}\Big\}.$$

Proof. Observe that $\mathcal{D} = \sum_{i=1}^{L-k+1} D_i$, where each D_i is an indicator random variable such that

$$\Pr[D_i = 1] = k\big(1 - p_s - p_d\big)^{k-1} p_d \frac{1}{(d+1)^{k-1}} = \hat{q}.$$

In simpler terms, $D_i = 1$ indicates that the k-span $\mathcal{K}_i$ has been introduced with only a single deletion, and no other mutation during the mutation process. The D_i's have the same dependence structure as the N_i's in the proof of Lemma 5, so proceeding in exactly the same fashion (simply replacing q by $\hat{q}$), the result follows. ◀

▶ **Lemma 7.** *Let $J_1 = \ln 2/\min\{d+1, 8\}$. Then, there exist absolute constants $c_1, c_2 > 0$ such that the following holds for any $\delta > 0$:*

$$\Pr\Big[|P - \mathbf{E}[P]| \geq 3\delta\Big] \leq 2\exp\Big\{-\frac{\delta^2}{8f_A}\Big\} + 2\exp\Big\{-\frac{\delta^2}{8(L - f_A)}\Big\} + 2\exp\Big\{-c_1\min\Big(\frac{\delta^2}{{c_2}^2{J_1}^2}, \frac{\delta}{c_2 J_1}\Big)\Big\}.$$

Proof. We can characterize $P = L' - 4f_A'$ by three separate groups of characters: the 'A' characters in S, the non-'A' characters in S, and the characters that are inserted into S by the mutation process.

First, we observe that an 'A' in S contributes -3 to P if it was untouched by the mutation process, contributes 1 to P if it was substituted into a non-'A' character by the mutation process, and contributes 0 to P if it was deleted by the mutation process. To capture these cases, we define X_i as follows:

$$X_i = \begin{cases} -3 & \text{with probability } 1 - p_s - p_d, \\ 1 & \text{with probability } p_s, \text{ and} \\ 0 & \text{with probability } p_d; \end{cases}$$

Next, we observe that a non-'A' character in S contributes 1 to P if it was untouched by the mutation process or substituted into another non-'A' character, contributes -3 to P if it is substituted into an 'A' character by the mutation process, and contributes 0 to P if it was deleted by the mutation process. To capture these cases, let

$$Y_i = \begin{cases} 1 & \text{with probability } 1 - p_s/3 - p_d, \\ -3 & \text{with probability } p_s/3, \text{ and} \\ 0 & \text{with probability } p_d; \end{cases}$$

Recall that C_i denotes the number of 'A's in Q_i, and $Z_i = l_i - 4C_i$. We can therefore express P in terms of the X_i's, Y_i's, and Z_i's as follows:

$$P = \sum_{i:S_i=\text{`A'}} X_i + \sum_{i:S_i\neq\text{`A'}} Y_i + \sum_{i=1}^{L} Z_i = X + Y + Z,$$

where for notational simplicity, we set $X = \sum_{i:S_i=\text{`A'}} X_i$, $Y = \sum_{i:S_i\neq\text{`A'}} Y_i$, and $Z = \sum_{i=1}^{L} Z_i$.

We first observe that

$$\begin{aligned} &\mathbf{E}[X_i] = p_s - 3(1 - p_s - p_d) = -3 + 4p_s + 3p_d, \\ &\mathbf{E}[Y_i] = 1 - \frac{p_s}{3} - p_d - p_s = 1 - \frac{4}{3}p_s - p_d, \quad \text{and} \\ &\mathbf{E}[Z_i] = 0; \end{aligned}$$

in the last equality we use the fact that $\mathbf{E}[l_i] = d$ and $\mathbf{E}[C_i] = d/4$; see (17). From these, we obtain

$$\begin{aligned} &\mathbf{E}[X] = f_A(-3 + 4p_s + 3p_d), \\ &\mathbf{E}[Y] = (L - f_A)\Big(1 - \frac{4}{3}p_s - p_d\Big), \\ &\mathbf{E}[Z] = 0, \quad \text{and} \\ &\mathbf{E}[P] = (L - 4f_A)\Big(1 - \frac{4}{3}p_s - p_d\Big). \end{aligned}$$

By Hoeffding's inequality (see Lemma 11), for any $\delta > 0$:

$$\Pr(|X - \mathbf{E}[X]| \geq \delta) \leq 2\exp\left\{-\frac{\delta^2}{8f_A}\right\}. \tag{19}$$

and

$$\Pr(|Y - \mathbf{E}[Y]| \geq \delta) \leq 2\exp\Big\{-\frac{\delta^2}{8(L - f_A)}\Big\}. \tag{20}$$

To show that Z is strongly concentrated around its mean, we first prove that the Z_i's are sub-exponential random variables and then use Lemma 13.

▶ **Lemma 14.** *Let* $J_1 = \ln 2/\min\{\frac{1}{d+1}, \frac{1}{8}\}$. *The following holds for each* Z_i *for all* $t \geq 0$*:*

$$\Pr[|Z_i| \geq t] \leq 4\exp\Big\{-t/J_1\Big\}.$$

We prove this lemma shortly, but first we finish the proof of Lemma 7.

As Z_i's are mean-zero sub-exponential random variables, it follows from Bernstein's inequality (Lemma 13) that there exist absolute constants $c_1, c_2 > 0$ such that the following holds for any $\delta > 0$

$$\Pr\Big[|Z| \geq \delta\Big] \leq 2\exp\left\{-c_1 \min\left(\frac{\delta^2}{{c_2}^2{J_1}^2}, \frac{\delta}{c_2 J_1}\right)\right\} \tag{21}$$

The triangle inequality and a union bound over (19), (20) and (21) completes the proof of Lemma 7. ◀

Proof of Lemma 14. Recall that $Z_i = l_i - 4C_i$, where l_i is a Geometric random variable with parameter $p = 1/(d+1)$ and mean d, and C_i is a Binomial random variable with parameters l_i and $1/4$. Let B_y be a Binomial random variable with parameters y and $1/4$, For $t \geq 0$, conditioning on the value of l_i and using Hoeffding's inequality, we obtain:

$$\begin{aligned}\Pr[|Z_i| \geq t] &= \sum_{y\geq 0} \Pr[l_i = y]\Pr[|B_y - y/4| \geq t/4] \\ &\leq 2p\sum_{y\geq 0}(1-p)^y \exp\left\{\frac{-t^2}{8y}\right\} \\ &\leq 2p\left(\sum_{y=0}^{\lfloor t\rfloor}(1-p)^y\exp\left\{-\frac{t^2}{8y}\right\} + \sum_{y=\lfloor t\rfloor+1}^{\infty}(1-p)^y\exp\left\{-\frac{t^2}{8y}\right\}\right).\end{aligned}$$

For $y \leq t$, we have $\frac{t^2}{8y} \geq \frac{t}{8}$, so $\exp\{-\frac{t^2}{8y}\} \leq \exp\{-t/8\}$ and we have

$$\sum_{y=0}^{\lfloor t\rfloor}(1-p)^y\exp\{-\frac{t^2}{8y}\} \leq \exp\{-t/8\}\sum_{y=0}^{\lfloor t\rfloor}(1-p)^y \leq \frac{e^{-t/8}}{p}.$$

When $y > t$,

$$\sum_{y=\lfloor t\rfloor+1}^{\infty}(1-p)^y\exp\{-\frac{t^2}{8y}\} \leq \sum_{y=\lfloor t\rfloor+1}^{\infty}(1-p)^y \leq \frac{(1-p)^t}{p} \leq \frac{e^{-pt}}{p}.$$

Putting these bounds together:

$$\Pr[|Z_i| \geq t] \leq 2\left(e^{-t/8} + e^{-pt}\right) \leq 4e^{-t/J_1}.$$

where $J_1 = \ln 2/\min\{p, 1/8\}$. ◀

▶ **Lemma 8.** *For any* $\delta \in (0,1)$*:*

$$\Pr\left[\Big|L' - \mathbf{E}[L']\Big| \leq \delta(Ld + Lp_d)\right] \geq 2\exp\left\{-\frac{L\delta^2 d^2}{2(d+1-\delta d)(d+1)}\right\} + 2\exp\left\{-\frac{Lp_d\delta^2}{3}\right\}.$$

Proof. We prove that L' is strongly concentrated around its expected value by proving concentration bounds for the individual pieces of (16). Specifically, we prove that $\sum l_i$ and $\sum D_i$ are strongly concentrated around their respective expected values.

For the sake of notational simplicity, let us define $\mathcal{L}_{\text{ins}} = \sum_{i=1}^{L} l_i$, and $\mathcal{L}_{\text{del}} = \sum_{i=1}^{L} D_i$. Then, L' is simply

$$L' = L + \mathcal{L}_{\text{ins}} - \mathcal{L}_{\text{del}}. \tag{22}$$

We first prove a strong concentration of $\mathcal{L}_{\text{ins}}$ around its expected value, and later prove the same result for $\mathcal{L}_{\text{del}}$. We finally use these results to show that L' is strongly concentrated around its mean.

We observe that every l_i is sampled from a geometric distribution with mean $d \geq 0$. This geometric distribution has support for values 0, 1, 2, 3, etc. (opposed to supporting positive values only). Another interpretation of this Geometric distribution is that it assigns probability to the number of failures before encountering a success – where each trial is a Bernoulli trial with success probability $1/(d+1)$. We also observe that the expected value of l_i is d, and therefore, the expected value of $\mathcal{L}_{\text{ins}}$ is Ld.

We aim to determine $\Pr[\mathcal{L}_{\text{ins}} \geq (1+\delta)Ld]$. We notice that $\mathcal{L}_{\text{ins}} \geq (1+\delta)Ld$ is equivalent to observing at least $(1+\delta)Ld$ failures before observing L successes from a series of Bernoulli trials with success probability $1/(d+1)$. Using the same argument, this is equivalent to observing at most L successes in $(1+\delta)Ld + L$ Bernoulli trials with success probability $1/(d+1)$. Let Z_i be a Bernoulli random variable with success probability $1/(d+1)$ for $i \geq 1$. Then, we have the following.

$$\Pr[\mathcal{L}_{\text{ins}} \geq (1+\delta)Ld] = \Pr[\sum_{i=1}^{(1+\delta)Ld+L} Z_i \leq L] \tag{23}$$

The expected number of success events in this collection of Bernoulli trials is $\mu = \frac{(1+\delta)Ld+L}{d+1}$. By reorganizing (23), we get the following result.

$$\Pr[\mathcal{L}_{\text{ins}} \geq (1+\delta)Ld] = \Pr[\sum_{i=1}^{(1+\delta)Ld+L} Z_i \leq (1-\epsilon)\mu] \tag{24}$$

where $\epsilon = \frac{\delta d}{\delta d + d + 1}$. Using Chernoff inequality for the sum of independent Bernoulli random variables from (13), the probability in (24) is at most $\exp\{-\epsilon^2\mu/2\}$. Using the values of μ and ϵ as defined before, we have the following result.

$$\Pr[\mathcal{L}_{\text{ins}} \geq (1+\delta)Ld] \leq \exp\{-\frac{L\delta^2 d^2}{2(d+1+\delta d)(d+1)}\} \tag{25}$$

Proceeding in an analogous manner, we can prove the probability for the other tail.

$$\Pr[\mathcal{L}_{\text{ins}} \leq (1-\delta)Ld] \leq \exp\{-\frac{L\delta^2 d^2}{2(d+1-\delta d)(d+1)}\} \tag{26}$$

Taking a union bound using the probabilities in (25) and (26), we have the following.

$$\Pr[|\mathcal{L}_{\text{ins}} - Ld| \leq \delta Ld] \geq 1 - 2\exp\{-\frac{L\delta^2 d^2}{2(d+1-\delta d)(d+1)}\} \tag{27}$$

We next prove that $\mathcal{L}_{\text{del}}$ is strongly concentrated around its mean. We recall that $\mathcal{L}_{\text{del}} = \sum_{i=1}^{L} D_i$, where each D_i for $i = 1$ to L is a Bernoulli trial with success probability p_d. Here, all D_i's are independent and identically distributed, and $\mathbf{E}[\mathcal{L}_{\text{del}}] = Lp_d$. Therefore, we can use the Chernoff bound in (14) for $\mathcal{L}_{\text{del}}$, which gives us the following result.

$$\Pr[|\mathcal{L}_{\text{del}} - Lp_d| \le \delta Lp_d] \;\ge\; 1 - 2\exp\{-\frac{Lp_d\delta^2}{3}\}. \tag{28}$$

Recall that $L' = L + \mathcal{L}_{\text{ins}} - \mathcal{L}_{\text{del}}$, as defined in (22). Therefore, by using the strong concentration of $\mathcal{L}_{\text{ins}}$ and $\mathcal{L}_{\text{del}}$ around their respective expected values in (27) and (28), the proof is completed. ◀

▶ **Theorem 9.** *Suppose $4f_A < L$ and $\frac{4}{3}p_s + p_d < 1$. Then, for sufficiently small $\delta > 0$, there exists constants $c_1, c_2 > 0$ such that*

$$\Pr[|\hat{p}_s - p_s| \ge 12\delta] \le 8k\exp\Big\{-\frac{\delta^2\mathbf{E}[\mathcal{N}]}{3k}\Big\} + 6k\exp\Big\{-\frac{\delta^2\mathbf{E}[\mathcal{D}]}{3k}\Big\} + 2\exp\Big\{-\frac{\delta^2\mathbf{E}[P]^2}{72f_A}\Big\}$$
$$+ 2\exp\Big\{-\frac{\delta^2\mathbf{E}[P]^2}{72(L-f_A)}\Big\} + 2\exp\Big\{-c_1\min\Big(\frac{\delta^2\mathbf{E}[P]^2}{{c_2}^2}, \frac{\delta\mathbf{E}[P]}{c_2}\Big)\Big\}.$$

Proof. Assume $4f_A < L$ and that $\frac{4}{3}p_s + p_d \le 1$. Recall that $P = L' - 4f_A'$ and let $Q = k\mathcal{N} + \mathcal{D}$, $R = k(L - 4f_A)\mathcal{N}$, and $T = 4k\mathcal{N} + \mathcal{D}$. Observe that

$$p_s = -3 \cdot \frac{\mathbf{E}[P]\mathbf{E}[Q] - \mathbf{E}[R]}{(L-4f_A)\mathbf{E}[T]}, \text{ and } \quad \hat{p}_s = -3 \cdot \frac{PQ - R}{(L-4f_A)T};$$

our proof will derive a concentration bound for $\hat{p}_s$ from those for the random variables P, Q, R, and T. First, by Lemma 5

$$\Pr[|\mathcal{N} - \mathbf{E}[\mathcal{N}]| \ge \delta\mathbf{E}[\mathcal{N}]] \le 2k\exp\Big\{-\frac{\delta^2\mathbf{E}[\mathcal{N}]}{3k}\Big\}.$$

So, since $4f_A < L$, we can multiply the inequality inside the probability by $k(L - 4f_A)$ to obtain that:

$$\Pr[|R - \mathbf{E}[R]| \ge \delta\mathbf{E}[R]] \le 2k\exp\Big\{-\frac{\delta^2\mathbf{E}[\mathcal{N}]}{3k}\Big\}.$$

From Lemmas 5 and 6, the triangle inequality and a union bound, we have that for any $\delta \ge 0$

$$\Pr[|Q - \mathbf{E}[Q]| \ge \delta\mathbf{E}[Q]] \le 3k\exp\Big\{-\frac{\delta^2\mathbf{E}[\mathcal{N}]}{3k}\Big\} + 3k\exp\Big\{-\frac{\delta^2\mathbf{E}[\mathcal{D}]}{3k}\Big\},$$
$$\Pr[|T - \mathbf{E}[T]| \ge \delta\mathbf{E}[T]] \le 3k\exp\Big\{-\frac{\delta^2\mathbf{E}[\mathcal{N}]}{3k}\Big\} + 3k\exp\Big\{-\frac{\delta^2\mathbf{E}[\mathcal{D}]}{3k}\Big\}.$$

Moreover, since under the assumption that $\frac{4}{3}p_s - p_d < 1$

$$\mathbf{E}[P] = (L - 4f_A)\Big(1 - \frac{4}{3}p_s - p_d\Big) > 0,$$

Lemma 7 implies there exists constants $c_1 > 0$ and $c_2 = c_2(d) > 0$ such that

$$\Pr\Big[|P - \mathbf{E}[P]| \ge \delta\mathbf{E}[P]\Big] \le 2\exp\Big\{-\frac{\delta^2\mathbf{E}[P]^2}{72f_A}\Big\} + 2\exp\Big\{-\frac{\delta^2\mathbf{E}[P]^2}{72(L-f_A)}\Big\} + 2\exp\Big\{-c_1\min\Big(\frac{\delta^2\mathbf{E}[P]^2}{{c_2}^2}, \frac{\delta\mathbf{E}[P]}{c_2}\Big)\Big\}.$$

Now, let us assume that the concentration bounds above do hold and that indeed:

$$(1-\delta)\,\mathbf{E}[P] \le P \le (1+\delta)\,\mathbf{E}[P], \tag{29}$$
$$(1-\delta)\,\mathbf{E}[Q] \le Q \le (1+\delta)\,\mathbf{E}[Q],$$
$$(1-\delta)\,\mathbf{E}[R] \le R \le (1+\delta)\,\mathbf{E}[R], \tag{30}$$
$$(1-\delta)\,\mathbf{E}[T] \le T \le (1+\delta)\,\mathbf{E}[T]. \tag{31}$$

Note that $\mathbf{E}[P], \mathbf{E}[Q], \mathbf{E}[R], \mathbf{E}[T] > 0$. Then,

$$\frac{(1-\delta)^2}{1+\delta}\frac{\mathbf{E}[P]\mathbf{E}[Q]}{\mathbf{E}[T]} \le \frac{PQ}{T} \le \frac{(1+\delta)^2}{1-\delta}\frac{\mathbf{E}[P]\mathbf{E}[Q]}{\mathbf{E}[T]}.$$

Observe that $\frac{(1-\delta)^2}{1+\delta} \ge 1-4\delta$ and $\frac{(1+\delta)^2}{1-\delta} \le 1+4\delta$ for small enough δ. Then, we have the following:

$$(1-4\delta)\frac{\mathbf{E}[P]\mathbf{E}[Q]}{\mathbf{E}[T]} \le \frac{PQ}{T} \le (1+4\delta)\frac{\mathbf{E}[P]\mathbf{E}[Q]}{\mathbf{E}[T]}. \tag{32}$$

Similarly, by dividing (30) by (31), and noting that that $\frac{1-\delta}{1+\delta} \ge 1-3\delta$, and $\frac{1+\delta}{1-\delta} \le 1+3\delta$ for small enough δ, we obtain:

$$(1-3\delta)\frac{\mathbf{E}[R]}{\mathbf{E}[T]} \le \frac{1-\delta}{1+\delta}\cdot\frac{\mathbf{E}[R]}{\mathbf{E}[T]} \le \frac{R}{T} \le \frac{1+\delta}{1-\delta}\cdot\frac{\mathbf{E}[R]}{\mathbf{E}[T]} \le (1+3\delta)\frac{\mathbf{E}[R]}{\mathbf{E}[T]}.$$

Combined with (32) this yields that

$$\frac{PQ-R}{T} \le (1+4\delta)\frac{\mathbf{E}[P]\mathbf{E}[Q]}{\mathbf{E}[T]} - (1-3\delta)\frac{\mathbf{E}[R]}{\mathbf{E}[T]} \le \frac{\mathbf{E}[P]\mathbf{E}[Q]-\mathbf{E}[R]}{\mathbf{E}[T]} + 4\delta\frac{\mathbf{E}[P]\mathbf{E}[Q]+\mathbf{E}[R]}{\mathbf{E}[T]}$$

and

$$\frac{PQ-R}{T} \ge \frac{\mathbf{E}[P]\mathbf{E}[Q]-\mathbf{E}[R]}{\mathbf{E}[T]} - 4\delta\frac{\mathbf{E}[P]\mathbf{E}[Q]+\mathbf{E}[R]}{\mathbf{E}[T]}.$$

Multiplying by $\frac{-3}{L-4f_A}$ we obtain that $p_s - 4\delta W \le \hat{p}_s \le p_s + 4\delta W$ with $W = 3\frac{\mathbf{E}[P]\mathbf{E}[Q]+\mathbf{E}[R]}{(L-4f_A)E[T]}$. It can be checked that $W \le 3$, and the result then follows from a union bound so that (29)-(31) all hold. ◀

E Interesting but unused results

Herein, we describe some interesting observations and results that were uncovered in the process of finding our main results, but which ultimately were not used in any proofs or the like. These include additional statistics, concentration inequalities, and an alternate, polynomial-based mutation parameter estimation approach.

E.1 Estimating the mutation rates using a polynomial

Unlike the estimators shown in Section 4, we can estimate the mutation rates by using a different combination of observations. In this section, we assume that $\mathbf{E}[\mathcal{S}]$, $\mathbf{E}[\mathcal{D}]$, and $\mathbf{E}[\mathcal{I}]$ are all positive. This is equivalent to assuming that p_s, p_d, and d are all non-zero. We later handle the cases for $p_s = 0$, and/or $p_d = 0$, and/or $d = 0$ separately.

For the ease of algebraic manipulations, we first define the following.

$$\mathcal{S}_{\text{norm}} = \frac{\mathcal{S}}{k\,\mathcal{K}}$$

$$\mathcal{I}_{\text{norm}} = \frac{\mathcal{I}}{(k-1)\mathcal{K}}$$

$$\mathcal{D}_{\text{norm}} = \frac{\mathcal{D}}{k\,\mathcal{K}}$$

We first take the ratio of (3) and (4) to get the following.

$$p_s = \frac{\mathbf{E}[\mathcal{S}_{\text{norm}}]}{\mathbf{E}[\mathcal{D}_{\text{norm}}]} p_d$$

We also take the ratio of (4) and (5) to derive the following.

$$\frac{\mathbf{E}[\mathcal{D}_{\text{norm}}]}{\mathbf{E}[\mathcal{I}_{\text{norm}}]} = \frac{d+1}{d} \times \frac{p_d}{1 - \mathbf{E}[\mathcal{S}_{\text{norm}}]p_d/\mathbf{E}[\mathcal{D}_{\text{norm}}] - p_d}$$

By reorganizing, we get the following.

$$d+1 = \frac{\mathbf{E}[\mathcal{D}_{\text{norm}}] - (\mathbf{E}[\mathcal{S}_{\text{norm}}] + \mathbf{E}[\mathcal{D}_{\text{norm}}])p_d}{\mathbf{E}[\mathcal{D}_{\text{norm}}] - (\mathbf{E}[\mathcal{S}_{\text{norm}}] + \mathbf{E}[\mathcal{D}_{\text{norm}}] + \mathbf{E}[\mathcal{I}_{\text{norm}}])p_d}$$

We next plug the values of $d+1$ and p_s into (4), and get the following polynomial after reorganizing.

$$\mathbf{E}[\mathcal{D}_{\text{norm}}]^k = p_d\Big(\mathbf{E}[\mathcal{D}_{\text{norm}}] - (\mathbf{E}[\mathcal{S}_{\text{norm}}] + \mathbf{E}[\mathcal{D}_{\text{norm}}] + \mathbf{E}[\mathcal{I}_{\text{norm}}])p_d\Big)^{k-1} \tag{33}$$

Given S and S', we can only observe $\mathcal{I}_{\text{norm}}$, $\mathcal{D}_{\text{norm}}$, and $\mathcal{I}_{\text{norm}}$. Using these observations, we can estimate p_d from (33).

$$\mathcal{D}_{\text{norm}}{}^k = \hat{p}_d\Big(\mathcal{D}_{\text{norm}} - (\mathcal{S}_{\text{norm}} + \mathcal{D}_{\text{norm}} + \mathcal{I}_{\text{norm}})\hat{p}_d\Big)^{k-1}$$

Let $y = \mathcal{D}_{\text{norm}} - (\mathcal{S}_{\text{norm}} + \mathcal{D}_{\text{norm}} + \mathcal{I}_{\text{norm}})\hat{p}_d$. This results in the following polynomial:

$$y^k - \mathcal{D}_{\text{norm}}\, y^{k-1} + (\mathcal{S}_{\text{norm}} + \mathcal{D}_{\text{norm}} + \mathcal{I}_{\text{norm}})\mathcal{D}_{\text{norm}}{}^k = 0,$$

where

$$\hat{p_d} = \frac{\mathcal{D}_{\text{norm}} - y}{\mathcal{S}_{\text{norm}} + \mathcal{D}_{\text{norm}} + \mathcal{I}_{\text{norm}}}.$$

After solving for $\hat{p_d}$, we estimate the other two rates as follows.

$$\hat{p_s} = \frac{\mathcal{S}_{\text{norm}}}{\mathcal{D}_{\text{norm}}}\hat{p_d}$$

$$\hat{d} = \frac{(\mathcal{D}_{\text{norm}})\hat{p_d}}{\mathcal{D}_{\text{norm}} - (\mathcal{S}_{\text{norm}} + \mathcal{D}_{\text{norm}} + \mathcal{I}_{\text{norm}})\hat{p_d}}$$

We next handle the edge cases in which these equations do not work. Note that we assume that $1 - p_s - p_d > 0$. From (4), we see that $p_d = 0$ if and only if $\mathbf{E}[\mathcal{D}] = 0$. Therefore, if we observe $\mathcal{D} = 0$, we estimate $\hat{p_d} = 0$. Using a similar argument, if we observe $\mathcal{S} = 0$, then we estimate $\hat{p_s} = 0$. And finally, if we observe $\mathcal{I} = 0$, then we estimate $\hat{d} = 0$.

E.2 Concentration of $f_A{}'$ around $\mathbf{E}[f_A{}']$

We next prove that $f_A{}'$ is strongly concentrated around its expected value. Recall the definition of $f_A{}'$ defined in (18). For the sake of notational simplicity, we define the following.

$$\mathcal{A} = \sum_{i:S_i=\text{‘A’}} A_i\,, \tag{34}$$

$$\mathcal{B} = \sum_{i:S_i\neq\text{‘A’}} B_i\,, \text{ and}$$

$$C = \sum_{i=1}^{L} C_i,$$

where A_i, B_i, and C_i are the same as introduced in Section 3. Using these notations, $f_A{}'$ is simply

$$f_A{}' = \mathcal{A} + \mathcal{B} + C.$$

E.2.1 Strong concentration of $\mathcal{A}$ around $\mathbf{E}[\mathcal{A}]$

We note that the A_i's in (34) are iid Bernoulli random variables with success probability $1 - p_s - p_d$. We also note that number of ‘A’ characters in S is f_A. Therefore, we have the following.

$$\mathbf{E}[\mathcal{A}] = f_A(1 - p_s - p_d)$$

As the $\mathcal{A}$ is a sum of iid Bernoulli random variables, we can use the Chernoff bound in (14) for $\mathcal{A}$, which gives us the following result.

$$\Pr\left[\left|\mathcal{A} - \mathbf{E}[\mathcal{A}]\right| \le \delta f_A(1 - p_s - p_d)\right] \ \ge\ 1 - 2\exp\{-\frac{\delta^2 f_A(1 - p_s - p_d)}{3}\} \tag{35}$$

E.2.2 Strong concentration of $\mathcal{B}$ around $\mathbf{E}[\mathcal{B}]$

We next prove a concentration bound for the sum of B_i's, as defined in (18). We note that B_i's here are iid Bernoulli random variables, each with success probability of $p_s/3$. We also note that the number of characters in S that are not 'A' is $L - f_A$. Therefore, we have the following result.

$$\mathbf{E}[\mathcal{B}] = (L - f_A)\frac{p_s}{3}$$

As $\mathcal{B}$ is simply a sum of iid Bernoulli random variables, we can use the same Chernoff inequality in (14) for $\mathcal{B}$, which gives us the following result.

$$\Pr\left[\left|\mathcal{B} - \mathbf{E}[\mathcal{B}]\right| \leq \delta(L - f_A)\frac{p_s}{3}\right] \geq 1 - 2\exp\{-\frac{\delta^2(L - f_A)p_s}{9}\} \quad (36)$$

E.2.3 Strong concentration of C around $\mathbf{E}[C]$

We finally prove a concentration inequality for $\sum_{i=1}^{L} C_i$, as defined in (18). Recall the result in (27) – developed for a sum of iid geometric random variables, each with mean d. We prove a similar result for C by showing that each C_i for $i = 1$ to L is geometrically distributed with mean $d/4$.

We first recall that the probability mass function (PMF) for a geometric distribution with parameter p. If a random variable X follows a geometric distribution with probability parameter p where $0 < p \leq 1$, then the PMF is as follows:

$$\Pr[X = j] = p(1 - p)^j,$$

where $j = 0, 1, 2, 3, \cdots$, etc. The expected value of X is given by

$$\mathbf{E}[X] = \frac{1 - p}{p}.$$

We can express the PMF of X using its mean. If the mean is m, then we need $p = 1/(m + 1)$, and therefore, the PMF of a geometric random variable with mean m is given by the following:

$$\Pr[X = j] = \frac{m^j}{(m + 1)^{j+1}}, \quad (37)$$

where $j = 0, 1, 2, 3, \cdots$, etc.

We next observe that C_i is the number of 'A' characters in Q_i, where Q_i is the string inserted after S_i and before S_{i+1}. The number of characters in Q_i is l_i, which follows a geometric distribution with mean d, and each character can be an 'A' with probability 1/4. Note that it immediately follows that $\mathbf{E}[C_i] = d/4$. We can therefore compute the probability that $C_i = j$ as follows:

$$\begin{aligned}
\Pr[C_i = j] &= \sum_{x=j}^{\infty} \Pr[C_i = j \mid l_i = x] \Pr[l_i = x] \\
&= \sum_{x=j}^{\infty} \binom{x}{j} \left(\frac{1}{4}\right)^j \left(\frac{3}{4}\right)^{x-j} \Pr[l_i = x] \\
&= \sum_{x=j}^{\infty} \binom{x}{j} \left(\frac{1}{4}\right)^j \left(\frac{3}{4}\right)^{x-j} \frac{d^x}{(d+1)^{x+1}} \\
&= \left(\frac{1}{4}\right)^j \left(\frac{4}{3}\right)^j \frac{1}{d+1} \sum_{x=j}^{\infty} \binom{x}{j} \left(\frac{3}{4}\right)^x \frac{d^x}{(d+1)^x} \\
&= \left(\frac{1}{4}\right)^j \left(\frac{4}{3}\right)^j \frac{1}{d+1} \sum_{x=j}^{\infty} \binom{x}{j} \left(\frac{3d}{4d+4}\right)^x \qquad (38)
\end{aligned}$$

We can now use the following summation for Binoamial coefficients:

$$\sum_{x=0}^{\infty} \binom{x}{j} r^x = \frac{r^j}{(1-r)^{j+1}},$$

where $|r| < 1$. In our case, $r = 3d/(4d+4)$. Our mutation process assumes $d \geq 0$, which means $4d + 4 > 3d$, and therefore, this summation can be used to simplify (38).

$$\begin{aligned}
\Pr[C_i = j] &= \left(\frac{1}{4}\right)^j \left(\frac{4}{3}\right)^j \frac{1}{d+1} \left(\frac{3d}{4d+4}\right)^j \frac{1}{\left(1 - \frac{3d}{4d+4}\right)^{j+1}} \\
&= \left(\frac{1}{4}\right)^j \left(\frac{4}{3}\right)^j \frac{1}{d+1} \times \frac{3^j d^j}{4^j (d+1)^j} \times \frac{4^{j+1}(d+1)^{j+1}}{(d+4)^{j+1}} \\
&= \frac{4d^j}{(d+1)^{j+1}} \\
&= \frac{(d/4)^j}{\left(d/4+1\right)^{j+1}}
\end{aligned}$$

This is the same form as the PMF of a geometric distribution shown in (37). Since $\mathbf{E}[C_i] = d/4$, it follows that each C_i is a geometric random variable with mean $d/4$.

We can now use the result proved for sum of geometric random variables with mean d, as shown in (27). Note that $C = \sum_{i=1}^{L} C_i$, which means $\mathbf{E}[C] = Ld/4$, where each C_i follows a geometric distribution with mean $d/4$. Using the result in (27) gives us the following:

$$\Pr\left[\left|C - \frac{Ld}{4}\right| \leq \delta \frac{Ld}{4}\right] \geq 1 - 2\exp\left\{-\frac{L\delta^2 d^2}{2(d+4-\delta d)(d+4)}\right\}. \qquad (39)$$

E.2.4 Strong concentration of $f_A{}'$ around $\mathbf{E}[f_A{}']$

We can now use the concentration bounds in (35), (36), and (39) to take a union bound and prove the following.

$$\Pr\left[\left|f_A' - \mathbf{E}[f_A']\right| \leq \delta \mathbf{E}[f_A']\right] \leq$$
$$1 - 2\exp\left\{-\frac{\delta^2 f_A(1-p_s-p_d)}{3}\right\} - 2\exp\left\{-\frac{\delta^2 (L-f_A)p_s}{9}\right\} - 2\exp\left\{-\frac{L\delta^2 d^2}{2(d+4-\delta d)(d+4)}\right\}.$$
